# Evolutionary genetics of pulmonary anatomical adaptations in deep-diving cetaceans

**DOI:** 10.1186/s12864-024-10263-9

**Published:** 2024-04-04

**Authors:** Boxiong Guo, Yixuan Sun, Yuehua Wang, Ya Zhang, Yu Zheng, Shixia Xu, Guang Yang, Wenhua Ren

**Affiliations:** Jiangsu Key Laboratory for Bioaffiliationersity and Biotechnology, College of Life Sciences, Nanjing Normal University, 210023 Nanjing, China

**Keywords:** Decompression sickness, Lung fibrosis, Amino acid substitution, Molecular evolutionary analysis, Cetaceans

## Abstract

**Background:**

Cetaceans, having experienced prolonged adaptation to aquatic environments, have undergone evolutionary changes in their respiratory systems. This process of evolution has resulted in the emergence of distinctive phenotypic traits, notably the abundance of elastic fibers and thickened alveolar walls in their lungs, which may facilitate alveolar collapse during diving. This structure helps selective exchange of oxygen and carbon dioxide, while minimizing nitrogen exchange, thereby reducing the risk of DCS. Nevertheless, the scientific inquiry into the mechanisms through which these unique phenotypic characteristics govern the diving behavior of marine mammals, including cetaceans, remains unresolved.

**Results:**

This study entails an evolutionary analysis of 42 genes associated with pulmonary fibrosis across 45 mammalian species. Twenty-one genes in cetaceans exhibited accelerated evolution, featuring specific amino acid substitutions in 14 of them. Primarily linked to the development of the respiratory system and lung morphological construction, these genes play a crucial role. Moreover, among marine mammals, we identified eight genes undergoing positive selection, and the evolutionary rates of three genes significantly correlated with diving depth. Specifically, the *SFTPC* gene exhibited convergent amino acid substitutions. Through in vitro cellular experiments, we illustrated that convergent amino acid site mutations in SFTPC contribute positively to pulmonary fibrosis in marine mammals, and the presence of this phenotype can induce deep alveolar collapse during diving, thereby reducing the risk of DCS during diving.

**Conclusions:**

The study unveils pivotal genetic signals in cetaceans and other marine mammals, arising through evolution. These genetic signals may influence lung characteristics in marine mammals and have been linked to a reduced risk of developing DCS. Moreover, the research serves as a valuable reference for delving deeper into human diving physiology.

**Supplementary Information:**

The online version contains supplementary material available at 10.1186/s12864-024-10263-9.

## Background

Human oceanic exploration has identified several dive-related illnesses, such as decompression sickness (DCS) [1, 2] and gas embolism [3]. DCS, being the most prevalent, is closely linked to various diving methods, including breath-hold diving [4–6] and scuba diving [7, 8]. In scuba diving, DCS mainly arises from the formation of gas bubbles in body tissues and blood, a result of rapid decompression. As divers inhale compressed air or gas mixtures at depth, elevated pressure leads to increased dissolution of nitrogen in their tissues [7, 8]. Should decompression transpire too rapidly during ascent or descent, nitrogen may effuse from the solution and form bubbles. These bubbles may induce a spectrum of symptoms, such as joint pain, nerve damage, and in extreme cases, paralysis or unconsciousness [1, 2]. Research indicates that DCS is a significant cause of fatality among scuba divers, second only to drowning [7]. Conducting multiple scuba dives in a short period significantly increases the risk of DCS and other related decompression illnesses [7, 8]. Although breath-hold divers do not inhale compressed gas and typically spend less time underwater, repeated deep dives over short intervals still increase their susceptibility to DCS. The risk of DCS in breath-hold diving rises with deeper dives and shorter surface intervals [4–6]. Breath-hold diving is predominantly observed in the animal kingdom. Post-activity neuroimaging studies of breath-hold divers have revealed DCS, evidenced by brain lesions [4]. Additionally, human divers who repeatedly dive to depths of 30–40 m or exceed 200 m in a single dive face increased DCS risk due to elevated nitrogen levels [5, 6]. Parallel research on wildlife, particularly loggerhead sea turtles (*Caretta caretta*), combines anatomical and physiological approaches. This research has identified clinical and pathological signs consistent with DCS in several captured specimens [9, 10].

DCS has been observed in various animals and humans participating in activities with variable pressure conditions [4–8, 10]. Marine mammals, particularly whales, engage in deep diving and may exhibit symptoms of DCS that are directly related to human activities, such as fishing and naval sonar interference [11–13]. However, these symptoms are not reported in their natural diving behavior, and instances of DCS are rare [13]. The reduced risk of DCS during diving may be linked to a specific lung phenotype, as supported by anatomical and physiological evidence [13–20]. Studies indicate that the thorax and lungs of many marine mammals are highly compliant, which may be related to the deep alveolar collapse and reinflation during dives [15, 21]. Research suggests that alveolar collapse, a primary mechanism in deep-diving marine mammals, limits nitrogen absorption and thereby mitigates the risk of DCS [13–15, 17, 22–26]. In these mammals, the alveolar walls are notably thickened [22, 27] and enriched with elastic fibers, exhibiting significant compliance and capable of reaching very low residual volumes (RV) [13, 15]. This trait likely aids in achieving negative buoyancy at depth and facilitating alveolar collapse, allowing for minimal dead space volumes and substantial tidal volumes (TV) for effective ventilation [22]. High thoracic compliance in marine mammals is also associated with this process [21]. In contrast, in typical animal lungs, an abundance of elastic fibers and thickened alveolar walls is often linked to pulmonary fibrotic diseases [28–30]. In humans, abundant elastic fibers and thickened alveolar walls typically lead to reduced lung compliance, adversely affecting breathing by making the lungs less expandable and impacting gas exchange efficiency [31]. However, marine mammals, such as cetaceans, exhibit exceptionally high lung compliance despite having thickened alveolar walls and an abundance of elastic fibers, a seemingly similar phenotype [21, 22]. This suggests cetacean lungs have unique regulatory mechanisms for more effective gas exchange during dives, preventing diving-related diseases, especially DCS.

Studies on cetaceans reveal that their lungs collapse during dives and re-expand upon ascent [14, 15, 17, 22]. Marine mammals possess pulmonary surfactants with enhanced capabilities to reduce alveolar surface tension compared to other animals [22, 32, 33]. This enhanced function facilitates the re-expansion of collapsed alveoli during the ascent phase of diving, effectively preventing potential harm from lung squeeze. This indicates that marine mammal surfactants are optimized for their aquatic lifestyle, ensuring physiological safety and health during frequent deep dives and ascents [22, 32, 33]. The surfactant system, in conjunction with structural adaptations of the respiratory system, plays a pivotal role in protecting against both lung squeeze and pulmonary barotrauma, thereby maintaining the integrity of the respiratory function in these animals [22, 32, 33]. Recent advancements in marine mammal respiratory physiology have led to the formulation of the “*Selective Gas Exchange* hypothesis” [13, 14]. This hypothesis explicates the mechanisms by which cetaceans and other marine mammals minimize nitrogen (N2) absorption while diving. Featuring a unique lung architecture with collapsible alveolar regions, and through the alignment of pulmonary blood flow with these regions, cetaceans can selectively exchange oxygen (O2) and carbon dioxide (CO2), while minimizing N2 exchange [13, 14]. Consequently, this reduces the risk of DCS in whales during dives. Similar observations in other marine mammals, such as *Leptonychotes weddelli*, show alveolar collapse correlating with diving depth [23, 26]. This indicates that for deep-diving marine mammals, profound alveoli collapse may benefit oxygen and carbon dioxide exchange during dives, inhibiting nitrogen absorption and thereby reducing DCS risk [17, 19, 34]. Molecular studies have identified two genes, *MAP3K19* and *SEC14L3*, lost exclusively in Cetacea and primarily expressed in the lungs [24]. *MAP3K19*, found in bronchial epithelial cells, type II pneumocytes, and lung macrophages [35], is implicated in pulmonary fibrosis [35] and chronic obstructive pulmonary disease (COPD) [36]. Inhibiting *MAP3K19* in mice models has shown a reduction in fibrosis and collagen deposition, thus preventing pulmonary fibrosis [35]. Additionally, this inhibition significantly diminishes lung inflammation and airway destruction, facilitating elastic fiber production, which is beneficial for alveolar collapse during diving [36]. *SEC14L3*, expressed in ciliated airway cells and type II pneumocytes, is involved in producing pulmonary surfactant, which prevents alveolar collapse [37–39]. The loss of *SEC14L3* in cetaceans may affect the surfactant’s composition and anti-adhesive properties [24]. Based on the anatomical and genomic evidence presented, we propose that the lungs of marine mammals, characterized by a high density of elastic fibers and a notable degree of flaccidity, are adapted to achieve low RV. This adaptation facilitates negative buoyancy and promotes alveolar collapse at depth. Additionally, pulmonary surfactant plays a critical role in ascending from dives. It aids in the expansion of alveoli and mitigates the risk of pressure-induced lung injuries and reduces the incidence of DCS during dives [22].

The advancement of sequencing technologies and the increasing abundance of mammalian genomic data afford an unparalleled opportunity to explore the molecular mechanisms underlying the pulmonary morphological features of cetaceans, as well as their adaptations to mitigate the risk of DCS during prolonged dives. In this study, we utilized a functional genomics approach to assess the evolution of 42 genes across 45 mammalian species, encompassing both cetaceans with high-quality genomes and their terrestrial counterparts. By integrating bioinformatic analysis with in vitro functional experiments, this study offers insights into the divergences in lung morphology among marine mammals and the molecular mechanisms that mitigate the risk of DCS during dives. These findings enhance our comprehension of the anatomy and physiology related to DCS and shed light on the evolutionary adaptations of marine mammals.

## Methods and materials

### Species coverage and sequence acquisition

In our study, we investigated 45 mammalian species with high-quality sequencing and assembly, representing a wide range of classifications (include species with a pulmonary fibrosis phenotype and their close relatives). The selected species belong to different orders including: Cetartiodactyla (*Tursiops truncatus*, *Orcinus orca*, *Delphinapterus leucas*, *Lipotes vexillifer*, *Physeter catodon*, *Balaenoptera acutorostrata*, *Balaenoptera musculus*, *Globicephala melas*, *Lagenorhynchus obliquidens*, *Monodon monoceros*, *Neophocaena asiaeorientalis*, *Phocoena sinus*, *Bos taurus*, *Ovis aries*, *Sus scrofa*, *Bos mutus* and *Bos grunniens*), Perissodactyla (*Equus caballus* and *Equus asinus*), Carnivora (*Odobenus rosmarus*, *Phoca vitulina*, *Leptonychotes weddellii*, *Neomonachus schauinslandi*, *Callorhinus ursinus*, *Eumetopias jubatus*, *Zalophus californianus*, *Halichoerus grypus*, *Mirounga leonine*, *Canis familiaris*, *Ailuropoda melanoleuca* and *Ursus maritimus*), Chiroptera (*Rhinolophus ferrumequinum*), Eulipotyphla (*Erinaceus europaeus*), Proboscidea (*Loxodonta africana*), Sirenia (*Trichechus manatus*), Pilosa (*Choloepus didactylus*), Dasyuromorphia (*Sarcophilus harrisii*), Ornithorhynchidae (*Ornithorhynchus anatinus*), Primates (*Homo sapiens*, *Rhinopithecus roxellana* and *Rhinopithecus bieti*), Rodentia (*Mus musculus* and *Rattus norvegicus*), and Lagomorpha (*Oryctolagus cuniculus* and *Ochotona curzoniae*) (Table S2).

The gene set associated with pulmonary fibrosis disease was collected from several previously published articles [40–43], as well as a database using the keyword ‘pulmonary fibrosis’ (https://www.informatics.jax.org/) (Table S1). The protein-coding sequences (CDS) were downloaded from the NCBI database (https://www.ncbi.nlm.nih.gov/) (Table S3). In addition, custom perl scripts were used to perform BLASTn searches to further verify partially or unannotated CDS. The longest transcript was retained for each gene in this analysis. To obtain higher quality sequences, we performed multiple sequence alignments for each orthologous gene using MACSE v2 [44] combined with PRANK v.170427 [45]. The aligned sequences were then trimmed using Gblocks v0.91 [46] with default settings.

### Molecular evolutionary analysis

Comparisons of nonsynonymous (dN)/synonymous (dS) substitution ratios have become a useful means for quantifying the impact of natural selection on molecular evolution [47, 48]. We employed the CODEML model implemented in the PAML 4.7 [49] software to perform codon-based maximum likelihood estimation of ω values. Values of ω < 1, = 1, and > 1 correspond to purifying selection, neutral evolution, and positive selection, respectively [50]. Considering the influences of base transition/transversion rates and codon usage in molecular evolution [51], we employed likelihood ratio tests (LRTs) to compare the hypotheses within each pair of nested models. To account for multiple testing, we utilized the Benjamini-Hochberg method [52] and set a false discovery rate (FDR) cutoff of 0.05.

To identify REGs in cetaceans, the two-ratio model in PAML was employed [49–53]. The null hypothesis (one-ratio model) assumed that genes in all-mammals evolved with the same ω, while the alternative hypothesis set two different ω values for the foreground (cetacean) and background (other mammal) species. Genes with ω (cetaceans) > ω (other mammals) were considered REGs in cetaceans [50].

To test whether positively selected sites were limited to a specific lineage, we used branch-site model and free-ratio model in the CODEML program to analyze the all-mammals dataset. The branch-site model can detect positive selection at specific sites along a specific branch, in both the null hypothesis (Ma0) and alternative hypothesis (Ma), distinctions in ω values are permitted among foreground and background branches and within sites in each gene. Under the Ma0 model, all species and sites are assumed to have ω values that do not exceed 1, indicating the absence of positive selection signals. In contrast, the Ma model permits certain sites in the foreground branch (cetaceans) to have ω values greater than 1, indicating the presence of positively selected sites [54, 55]. Whereas the free-ratio model allows an independent ω value for each branch [49], we compared the free-ratio model that assumes an independent ω ratio for each branch with the null one-ratio model with the same ω for all branches. When the value of dN or dS in each ω value is less than 0.0002, we considered it as an outlier “n/a” [56]. The relative goodness-of-fit of the nested models was determined using LRTs. All nested models were statistically different from the null model (*P* < 0.05) [57].

### Identification of specific and convergent amino acids

We used the Fasparser 2.0 [58] to detect specific amino acids in cetaceans and convergent amino acids in marine mammals, respectively. Human canonical sequences in UniProt (https://www.uniprot.org/) [59] were taken as references for the locations of amino acids.

### Mapping of positively selected sites and cetacean-specific sites onto protein structures

To investigate the impact of positively selected sites and cetacean-specific sites on protein function. We used Swiss-Model (https://swissmodel.expasy.org/) [60] to predict the 3D structure, then used the Pfam (http://pfam.xfam.org/) [61] website to search for domains of each protein. Finally, the EzMol (http://www.sbg.bio.ic.ac.uk/~ezmol/) [62] was used to annotate the positively selective sites and functional domains in the obtained 3D structure.

### Association analysis between gene evolution and diving depth

In order to investigate the potential relationship between the gene evolutionary rate (ω) and maximum diving depth, we utilized root-to-tip ω, which refers to the average ω value accumulated from the last common ancestor to the extant species and serves as a selection indicator encompassing the evolutionary history of the gene within a given species [63, 64]. The evolutionary rate of each gene was calculated using codeml in PAML [49] with a free-ratio model. The diving depth data of marine mammals were collected from previously published sources (Table S7). PGLS regression [65] was used to analyse the relationship between log (root-to-tip ω) and log (max-dive-depth m). The lambda (λ) value estimated by the maximum likelihood method was used as a quantitative measure of phylogenetic signals [66]. All statistical analyses were performed using R in the package Caper [67].

### Gene functions and signaling pathway annotation and enrichment

The genes were subjected to GO functional [68] enrichment analyses using the Metascape (http://metascape.org) [69]. In Metascape, we set *Homo sapiens* as the “Input as species” and “Analysis as species” and used a cutoff of *p*-value < 0.05.

### Cell culture, lentiviral production and infection

Human NSCLC cell line A549 cells was purchased from ATCC and cultured according to the protocols of the ATCC. All culture media were purchased from WISENT, cell culture dishes were purchased from Jet Biofil. The sequence of Whale-SFTPC was generated in Beijing Tsingke Biotech Co. Ltd. Lentiviral vectors (Lentivirus-mCherry-Puro-CMV-N3xFlag-BI-SFTPC/mutSFTPC, Lentivirus-mCherry-Puro-CMV-N3xFlag-Mouse-SFTPC/mutSFTPC) were produced by Corues Biotechnology Company (Nanjing, China), and lentiviral infection protocol followed the company’s instructions. The sequence is shown in the Table S11.

### DNA extraction and PCR amplification

Fresh mouse lung tissue samples were utilized, and RNA was extracted from these samples following the standard phenol-chloroform extraction protocol. Mice were euthanized by cervical dislocation and acquired from GemPharmatech Co., Ltd. (Nanjing, China). The cDNA of mouse SFTPC was amplified using PCR. PCR profiles were 95 °C/5 min, 95 °C–30 s, 70 °C–30 s, 72 °C–40 s, and 72 °C/5 min cycles, repeated 35 times. PCR products were directly sequenced using an ABI 3730 DNA sequencer (Sangon Biotech, Shanghai Co., Ltd.). The construction of mouse and cetacean SFTPC gene mutants with individual amino acid mutations (I123V, V123I) followed the instructions of the Beyotime (Shanghai, China) Quick Mutation kit. All primers used for amplification are listed in Table S10.

### Antibodies

Antibodies used were as follows: antibodies against Cleaved-Caspase 3 (cat# 9664), was purchased from Cell Signaling Technology, Inc., Danvers, MA, USA; antibodies against MMP7 (cat# A20701), ACTA2 (cat# A17910) were purchased from ABclonal (Wuhan, China); antibodies against SFTPC (cat#10774-1-AP) and beta-Tubulin (cat# 10094-1-AP) were purchased from Proteintech Group, Rosemont, IL, USA.

### Western blotting

Collect the cells and lyse them in cell lysis buffer (50 mM Tris·HCl, pH 7.5, 150 mM NaCl, 1% NonidetP-40, 0.5% sodium deoxycholate, and 1% protease inhibitor cocktails, MCE), centrifuge the cell lysate at 4 °C, 14,000 rpm for 15 min, In Western blot studies, protein concentrations of samples were determined using the Bradford protein method, and 20 µg of proteins for each sample were separated by SDS-PAGE followed by transfer to polyvinylidene difluoride (PVDF) membranes. The membranes were blocked for 1 h at room temperature with 5% nonfat milk in the TBST buffer and incubated with a specific primary antibody diluted in TBST buffer overnight at 4 ℃. After extensive washes by TBST buffer, the membranes were incubated with a corresponding secondary antibody for 1 h at room temperature, and the immunoreactive bands were visualized by using an ECL kit (Vazyme Biotech Co. Ltd., Nanjing, China).

### Cell viability assay

Cell proliferation was assessed through CCK8 assays. Cells were briefly seeded at 3 × 103 cells/well in triplicate in a 96-well plate and cultured overnight. At 24 h and 48 h, 10 µL of CCK8 solution (Vazyme Biotech Co. Ltd., Nanjing, China) was added to each well, followed by incubation at 450 nm for an additional 2 h using a microplate reader to measure absorbance. Cell viability for each treatment was determined as a percentage of its OD450 nm compared to the vehicle control, calculated using GraphPad Prism 8.0 software.

### Statistical analysis

Experiments were carried out at least 3 times unless otherwise stated. The software GraphPad Prism 8.0 was used for statistical analysis and the data are shown as mean ± S.D. Student’s t-test and one way ANOVA were employed to assess the statistical significance of differences between data sets. A *p*-value of lower than 0.05 was considered to be significant. *p* < 0.05 (*), *p* < 0.01 (**), and *p* < 0.001 (***).

## Results

### Rapidly evolving genes and positively selected genes in cetaceans

**Fig. 1:**
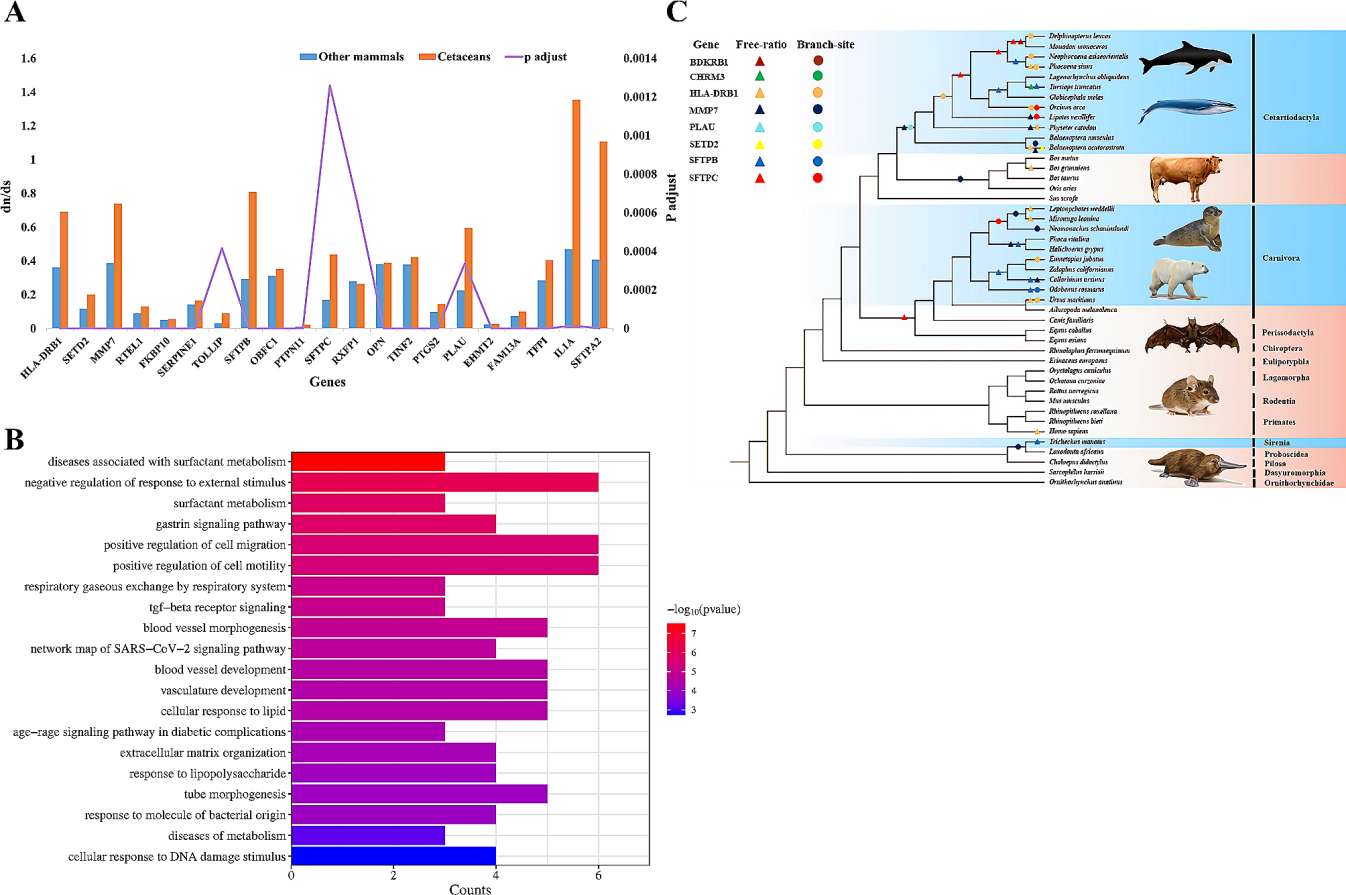
REGs and PSGs that were identified using PAML. **(A)** Twenty-one REGs in cetaceans with ω_cetaceans > ω_other mammals (adjusted *p*-value < 0.05). **(B)** GO enrichment terms for Twenty-one REGs in cetaceans. The 20 related terms in biological process are shown. **(C)** Evidence of positive selection across the phylogeny of mammals identified by the free-ratio and branch-site models. In the phylogenetic tree, blue represents marine mammals, and orange represents terrestrial mammals. Triangles and circles represent the free-ratio and branch-site models, respectively. The silhouette pictures are from the Internet

Positive selection and rapid evolution of specific genes result from the accumulation of genetic variation in response to environmental changes, a component of Biological Adaptation to Natural Selection [70]. In this study, we employed the Consensus Tree from TimeTree (Fig. S1) and the CODEML model in PAML to detect rapidly evolving genes (REGs) and positively selected genes (PSGs) across 45 mammalian species, designating cetaceans as the foreground species and other mammals as the background. Employing branch model analyses with CODEML, we identified 21 REGs in cetaceans, adjusting for multiple testing using the FDR method (adjusted *p*-value < 0.05) (Table S4). The evolutionary rates of these genes were notably higher in cetaceans than in other mammalian species (Fig. 1A), implying unique selective pressures on cetaceans. Enrichment analysis further disclosed that REGs in cetaceans were linked to respiratory gaseous exchange by the respiratory system, diseases associated with surfactant metabolism, blood vessel development, and blood vessel morphogenesis (Fig. 1B).

The Branch model (One-ratio vs. Free-ratio) and Branch-site models were utilized to identify positive selection in eight cetacean genes [71] (Table S5, Table S6). Four of these genes (*SFTPB*, *SFTPC*, *MMP7*, *HLA-DRB1*) exhibited positive selection in the background branch species as well (Fig. 1C). To establish the relationship between the proposed positively selected amino-acid sites and protein function, we conducted a search on the UniProt database to map these sites onto their respective 3D structures. The results revealed that certain positively selected amino-acid sites were located within crucial functional domains of proteins (Fig. S2). Specifically, site 40 and 69 of *MMP7* exhibit positive selection in the Putative peptidoglycan binding domain, and sites 104, 115, 121, 131 are situated in the Matrixin domain. Previous reports indicate that *MMP7* overexpression induces apoptosis of alveolar epithelial cells and migration of interstitial cells, leading to the destruction of the alveolar wall and the development of pulmonary fibrosis [43, 72] (Fig. S2).

### Specific amino acid substitutions in cetaceans

**Fig. 2:**
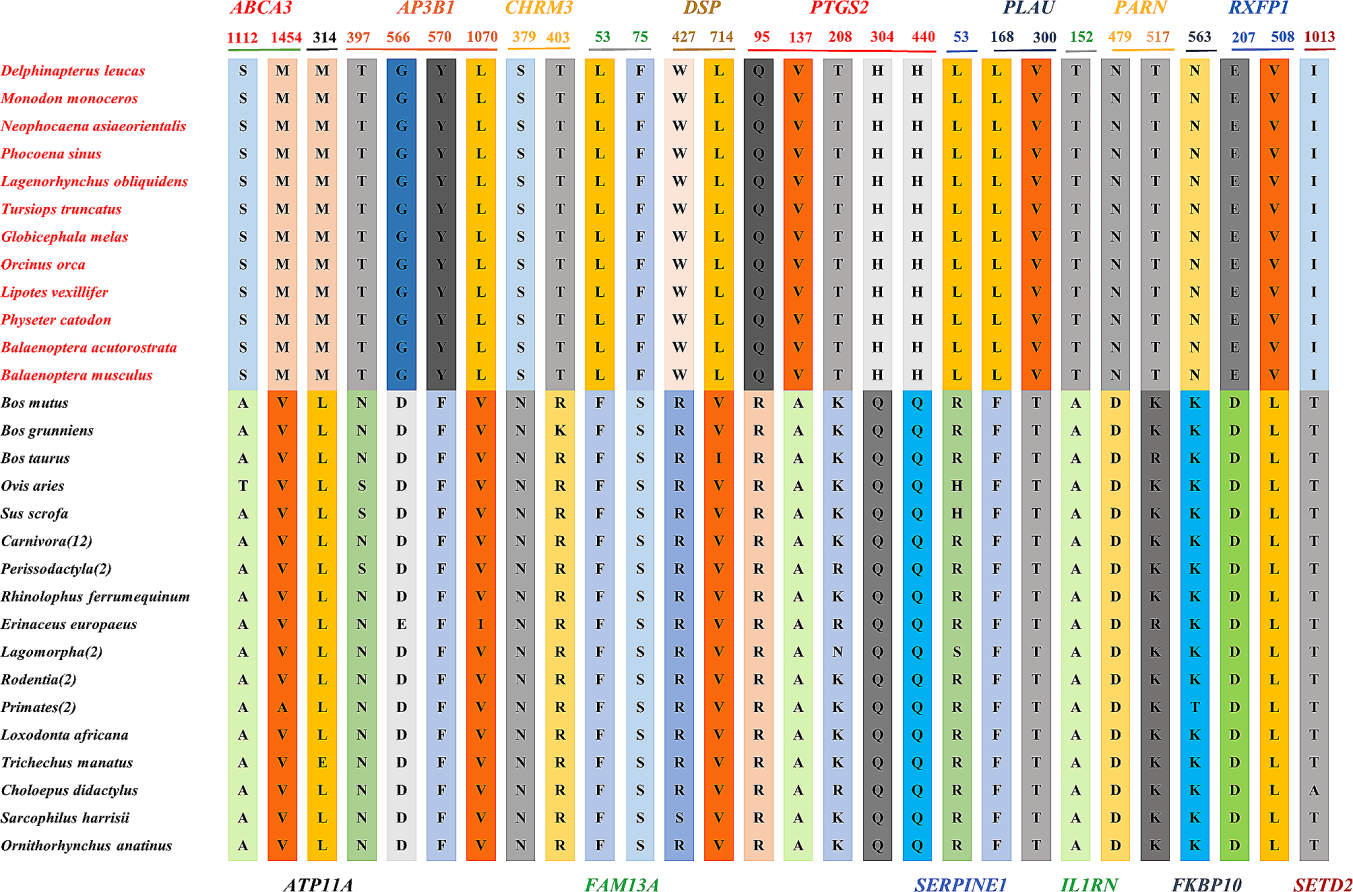
Specific amino acid substitutions in cetaceans. Fourteen proteins were identified to have undergone specific amino acid substitutions, indicated by red text for cetaceans and black text for other mammals. Different colors are used to represent different amino acids at the same position in the protein

Fixed amino acid replacements within a specific group of mammals may contribute to explaining a particular phenotype [56]. We detected 28 cetacean-specific amino acid substitutions in 14 proteins (Fig. 2). Enrichment analysis unveiled associations of the genes encoding these proteins with inflammatory response, lung development, and respiratory system development (Fig. S3). Mapping the cetacean-specific sites to their respective 3D structures was done to assess their functional relevance. The results revealed that certain sites were located within crucial functional domains of proteins (Fig. S4). As an example, the cetacean SERPINE1 protein exhibited a specific amino acid site (53) within the Serpin (serine protease inhibitor) domain. Previous reports indicate that mutations or functional defects in the Serpin domain may result in the loss of its ability to inhibit protease activity, contributing to its functional abnormality in pulmonary fibrosis [73, 74] (Fig. S4).

**Fig. 3:**
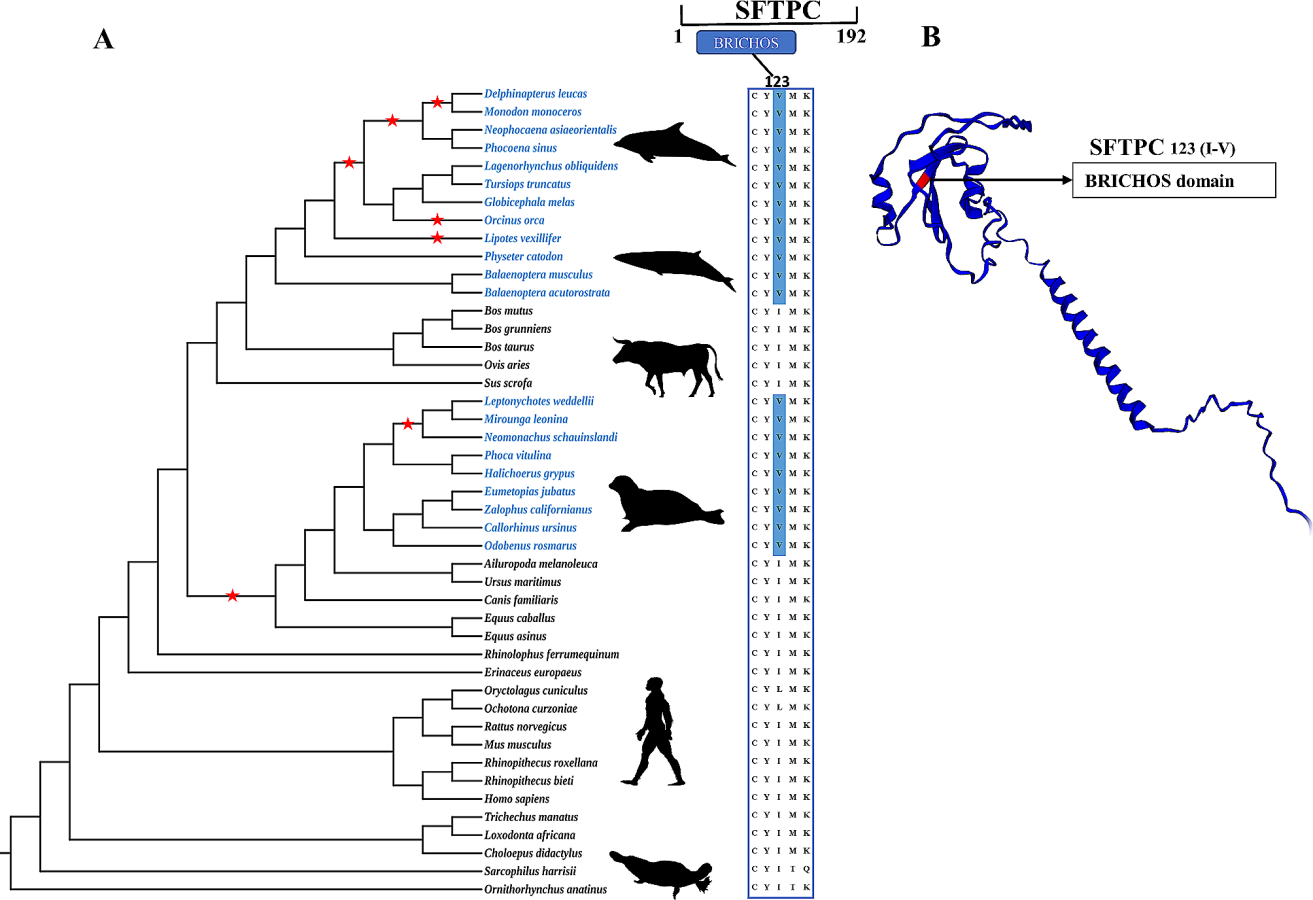
Convergent amino acid substitutions between cetaceans and other marine mammals. **(A)** The SFTPC protein consists of 192 amino acids. In marine mammals, the 123rd amino acid is mutated from isoleucine (I) to valine (V) and is located in the important functional domain BRICHOS of SFTPC protein. In the phylogenetic tree, blue font represents marine mammals excluding manatees, and black font represents terrestrial mammals. The red pentagram represents the lineage under positive selection. The silhouette pictures are from the PhyloPic (https://www.phylopic.org/). **(B)** The three-dimensional structure of SFTPC was built using Swiss-Model, and the red markings indicate convergent amino acid site in marine mammals, which are located in the BRICHOS domain

### Association between gene evolution and maximum diving depth

**Fig. 4:**
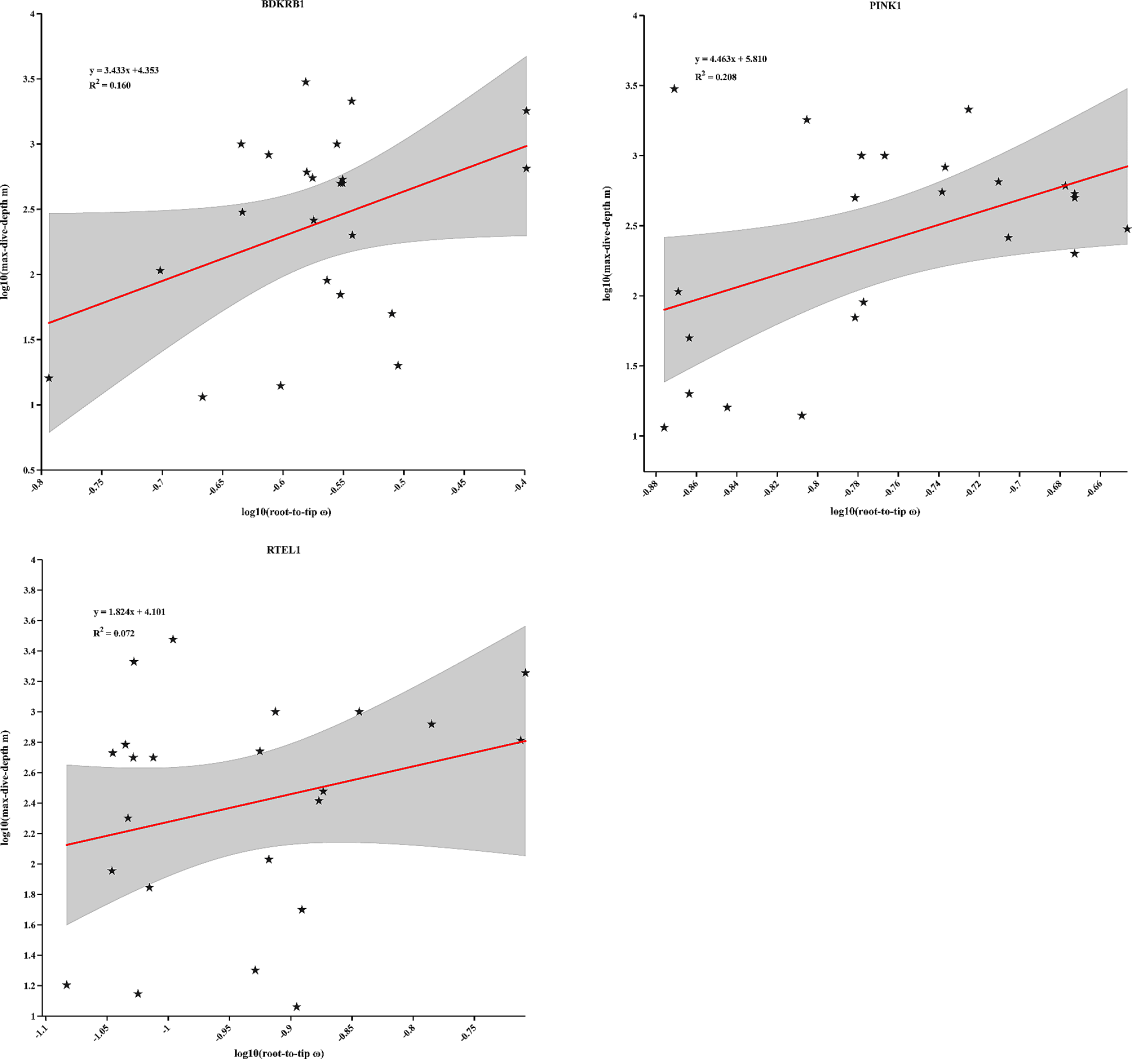
Regression analyses between root-to-tip ω and maximum diving depth. Scatterplots of significant relationships between log10 (max-dive-depth m) and log10(root-to-tip ω)

Evaluating the statistical association between genes and normal phenotypic variation is a well-regarded strategy for investigating the phenotypic implications of an ongoing selection signature [75]. Phylogenetic generalized least squares (PGLS) regressions were conducted to explicitly explore the connection between the evolutionary rate of genes and maximum diving depth. Regression analyses demonstrated a significant positive association between log10 (root-to-tip ω) and log10 (max-dive-depth m) in three genes, namely *BDKRB1* (R^2^ = 0.160, *p* = 0.0434), *PINK1* (R^2^ = 0.208, *p* = 0.0272), and *RTEL1* (R^2^ = 0.072, *p* = 0.0382) (Fig. 4, Table S7, Table S8).

### Functional convergence of the *SFTPC* among marine mammals

Initial observations revealed convergent/parallel amino acid substitutions in the SFTPC protein (SP-C) among 23 marine mammals (excluding *Trichechus manatus*). At position 123 of the SP-C, marine mammals exhibit Valine, while terrestrial mammals display Isoleucine (Fig. 3A). To ascertain the relationship between this convergent site and protein function, we mapped the site onto the three-dimensional structure of the SP-C. The results indicated that the site is situated in the BRICHOS domain of the SP-C (Fig. 3B).

### Correlation between *SFTPC* gene mutations and protein expression of pulmonary fibrosis markers

**Fig. 5:**
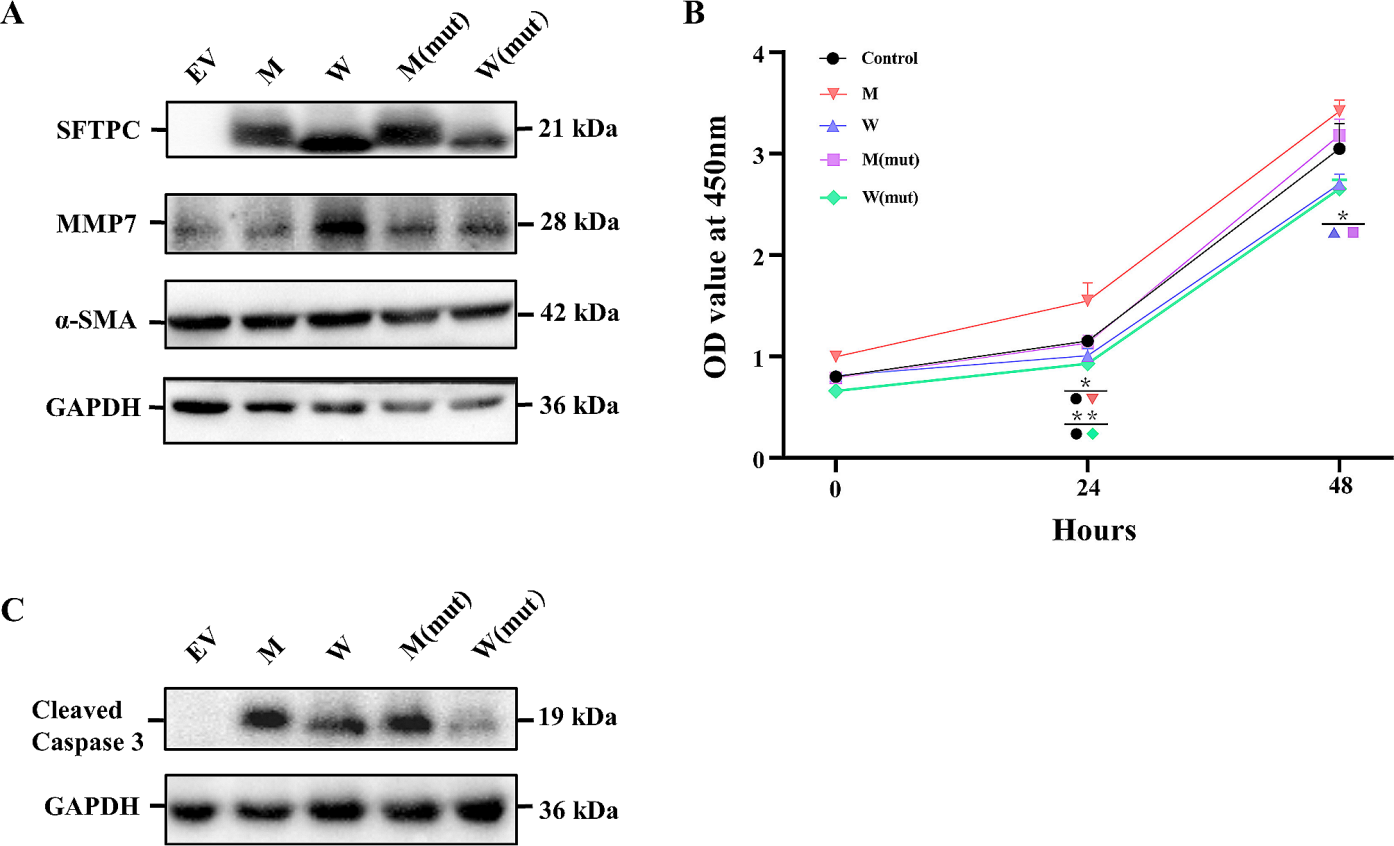
The convergent site of *SFTPC* gene enhances pulmonary fibrosis in marine mammals.*SFTPC* overexpression in A549 cell lines, Western bloy analyses of pulmonary fibrosis markers(**A**) and apoptotic marker(**C**). (**B**)CCK8 assays detected the effects of after overexpressing *SFTPC* on the proliferation of A549 cell lines. EV is Lev-mCherry-Puro- CMV-N3xFlag-control, M is Lev-mCherry-Puro-CMV-N3xFlag-Mouse-SFTPC, W is Lev- mCherry-Puro-CMV-N3xFlag-BI-SFTPC, M(mut) is Lentivirus-mCherry-Puro-CMV- N3xFlag-Mouse-mutSFTPC, W(mut) is Lentivirus-mCherry-Puro-CMV-N3xFlag-BI- mutSFTPC. Date repressen the mean ± S.D.*p < 0.05,**p < 0.01. The cell lysates were subjected to Western blot analyses using indicated antibodies with Tubulin as a loading control. The blots/gels displayed in the figure represent cropped versions, delineated by black dividing lines. Full-length blots/gels are presented in Fig S5

We investigated whether convergent evolutionary site variations in the *SFTPC* gene in marine mammals enhance pulmonary fibrosis, leading to alveolar collapse during diving and potentially reducing the risk of DCS. Various *SFTPC* variants were overexpressed, and western blotting techniques were utilized to assess whether variations in *SFTPC* gene loci induce alterations in the expression levels of fibrotic marker proteins, including MMP7 (Matrix Metalloproteinase 7) and α-SMA (Alpha-Smooth Muscle Actin). The results show that in whales, the expression levels of MMP7 and α-SMA proteins in wild-type individuals are higher than those in the control and mouse wild-type. Additionally, in whale mutants, there is a decrease in the expression of both MMP7 and α-SMA proteins compared to their wild-type counterparts. In mouse mutants, there is an increase in MMP7 protein expression relative to their wild-type, but no significant change in α-SMA protein expression (Fig. 5A).

### Correlation between *SFTPC* gene mutations and cell proliferation and apoptosis abilities

The regulatory mechanisms connecting pulmonary fibrosis and endoplasmic reticulum (ER) stress are intricately linked [76]. Subsequently, we conducted a thorough investigation to determine whether lung fibrosis resulting from *SFTPC* gene mutations influences cellular proliferation and apoptosis during the ER stress phase. Initially, we evaluated cell proliferation levels after overexpressing various *SFTPC* variants. Our results indicate that in cell proliferation assays, the five experimental groups exhibited higher cell proliferation capabilities at 24 and 48 h in both wild-type and mutant mice compared to wild-type and mutant whales. No significant changes were observed in proliferation capabilities between wild-type and mutant whales compared to the control and between wild-type and mutant mice (Fig. 5B, Table S9). We then analyzed alterations in the expression levels of the apoptotic effector, Cleaved-caspase 3, using protein assays. We observed that, compared to the control, the expression level of Cleaved-caspase 3 increased in wild-type whales, and it was higher in wild-type mice compared to wild-type whales (Fig. 5C).

## Discussion

Cetaceans are mammals that have completely transitioned from a terrestrial to an aquatic lifestyle, and their respiratory systems have undergone adaptive changes to cope with the process of breathing in the ocean. The lungs, among these adaptations, serve as a crucial interface between the body and the environment during respiration under low-oxygen conditions [77–79]. Research has demonstrated that the lungs of cetaceans, have undergone adaptive evolution related to resistance against oxidative stress [77, 80]. Understanding the molecular evolutionary mechanisms that drive diving adaptations in whales is essential for comprehending the treatment of diving-related disorders. Proposals suggest that the lungs of cetaceans have developed specific features, including a notable presence of elastic fibers, to adapt to diving and breath-holding [22]. From the perspective of molecular evolution, we found in this study that genes associated with pulmonary fibrosis have undergone adaptive evolution in marine mammals such as whales. This adaptation is essential for their adaptation to hypoxic environments and mitigation of DCS risk.

### The adaptation of cetaceans to diving and breath-holding is the result of coevolution of multiple genes

Our study results indicate accelerated evolution and specific amino acid substitutions in multiple genes related to lung fibrosis in cetaceans. Functional enrichment analysis revealed their main enrichment in processes such as respiratory gas exchange, cellular response to DNA damage stimuli, pulmonary development, and inflammatory response. This suggests that these genes not only contribute to lung fibrosis in cetaceans but are also closely linked to their adaptation to hypoxic environments, diving, and breath-holding. For example, the *RXFP1* gene encodes a receptor protein belonging to the relaxin/insulin-like peptide family receptor [81]. In a mouse experiment, *RXFP1* expression alleviated symptoms of lung fibrosis and pulmonary arterial hypertension [81, 82]. *RXFP1* serves as a receptor for relaxins, with its activity facilitated through G proteins. This interaction stimulates adenylate cyclase, leading to elevated cAMP levels [83]. The expression of *RXFP1* is directly related to the pulmonary function of patients with Idiopathic Pulmonary Fibrosis. Enhanced *RXFP1* expression is associated with reduced levels of α-SMA, a marker protein for pulmonary fibrosis, suggesting a potential role in fibrosis inhibition [81, 82]. We propose that *RXFP1* pathways or similar mechanisms may underlie the superior pulmonary function observed in marine mammals. These animals exhibit remarkable lung compliance, possibly due to *RXFP1*-mediated modulation of tissue remodeling and fibrosis [81, 82]. This regulatory process resembles the *MAP3K19*-induced inhibition of lung fibrosis, indicating potential regulatory overlaps in marine mammal lung fibrosis [24]. Such mechanisms could enable these lungs to remain highly compliant despite the presence of significant fibrous tissue. The *PLAU* gene encodes an enzymatic protein known as plasminogen activator [84]. Research has demonstrated the pivotal role of *PLAU* in hypoxia adaptation for cetaceans [85]. This study primarily employed a genome-wide large-scale screen, determining the association of the *PLAU* gene with hypoxia adaptation in porpoises and its likely prominent role in hypoxic responses [85, 86]. *PLAU* also promoted Esophageal squamous cell carcinoma proliferation and colony formation through MAPK pathway, and promoted migration through up-regulation of Slug and MMP9, leading to an increase in fibrosis [84]. Similarly, numerous genes play roles in lung development, such as *ABCA3*. The *AP3B1* gene encodes the β3A subunit of the adaptor protein complex 3 (AP-3), and mutations in the *AP3B1* gene result in Hermansky-Pudlak syndrome (HPS) subtypes HPS-2 and HPS-10 [87]. Pulmonary fibrosis develops in HPS-1, HPS-2, and HPS-4 [87, 88]. The findings indicate that the adaptive evolution of genes associated with pulmonary fibrosis may play a role in the development of diverse elastic fiber and thickening of alveolar walls phenotypes in cetacean lungs [22]. These findings enhance our comprehension of the variations in lung morphology and anatomy among marine mammals. They offer insights into the molecular mechanisms underlying breath-holding capabilities and the reduction of DCS risk in these animals.

### The high abundance of elastic fibers in the lungs is a common strategy among marine mammals to reduce the risk of DCS in hypoxic environments

The mechanisms marine mammals, including cetaceans, use to mitigate DCS risk during prolonged dives have been extensively investigated. Macroscopic evidence shows that their lungs, enriched with loosely distributed elastic fibers, enable a deep collapse during dives, reducing DCS risk. The primary support for this comes from anatomical studies [22]. However, there is a significant lack of molecular evidence from an evolutionary standpoint.

By comparing two models (branch model and branch-site model) using PAML, a prevalence of positive selection was found in lung fibrosis-related genes among marine mammals, with the majority of positively selected sites situated in crucial functional protein domains. In humans and other mammals, elastic fibers in the lungs constitute a minimal proportion [89], ensuring optimal elasticity and morphological stability for efficient gas exchange and respiratory function [89, 90]. While a thickened alveolar wall and a large number of elastic fibers in the lungs may induce lung fibrosis in typical mammals, resulting in respiratory challenges, marine mammals do not experience such issues. This phenomenon may be attributed to cetaceans’ possession of a dual capillary network in the lungs [22, 91], enhancing gas exchange between alveoli and capillary blood, averting respiratory distress despite fibrosis. Concerning the maintenance of high pulmonary compliance in marine mammals with this distinct phenotype [21], we propose the existence of supplementary regulatory mechanisms within the lungs that govern this process. Additionally, our research indicates that the evolution of the *RXFP1* gene may be pivotal in controlling pulmonary fibrosis, thus facilitating the preservation of high pulmonary compliance and significantly improving respiratory efficiency while diving [81, 82]. The elevated fibrosis in cetacean lungs suggests that lung fibrosis-related genes have undergone adaptive evolution in marine mammals to facilitate their diving and respiratory processes. Eight lung fibrosis-related genes were identified: *BDKRB1*, *CHRM3*, *HLA-DRB1*, *MMP7*, *PLAU*, *SETD2*, *SFTPB*, and *SFTPC*. *BDKRB1* encodes the bradykinin B1 receptor, binding to the peptide hormone bradykinin and engaging in various inflammatory response processes [92]. Studies have demonstrated that the deletion of *BDKRB1* in mice inhibits fibrosis [92], suggesting the receptor’s role in promoting fibrosis and its potential significance in mammalian lung fibrosis [92]. *SFTPB* and *SFTPC* genes encode vital components of pulmonary surfactant, crucial for regulating alveolar surface tension and maintaining lung tissue architecture [93, 94]. Mutations in the *CHRM3*, *HLA-DRB1*, *PLAU*, and *SETD2* genes are closely linked to the pathogenesis of pulmonary fibrosis [40, 86, 95, 96]. The *MMP7* gene, serving as a biomarker for pulmonary fibrosis, has been demonstrated to contribute to the development of lung fibrosis through its elevated expression [43, 72].

Simultaneously, correlation analysis reveals a positive relationship between the evolutionary rates of the *BDKRB1*, *PINK1*, and *RTEL1* genes and maximum diving depth. The functions of these genes are linked to the formation of pulmonary fibrosis, implying their significant role in the diving process of cetaceans. For example, research has demonstrated that mutations in the *RTEL1* gene contribute to an animal’s adaptation to hypoxic conditions [97]. In mice, the reduction of *PINK1* expression in lung epithelial cells resulted in mitochondrial depolarization and the expression of profibrotic factors [98]. The collective findings indicate that the adaptive evolution of genes linked to pulmonary fibrosis in marine mammals offers insights into the variations in their lung morphology and the associated risk of developing DCS due to lung collapse [22, 98].

### The evolution of *SFTPC* may have contributed to pulmonary fibrosis in marine mammals

Pulmonary surfactant, composed of lipids and proteins, is crucial for alveolar stability, surface tension reduction, and gas exchange in the lungs [32]. *SFTPC*, encoding a hydrophobin SP-C in type II alveolar epithelial cells, is integral to these functions [99]. The BRICHOS domain (residues 94–197) in SP-C prevents protein aggregation, ensuring proper folding and stabilization [100, 101]. The BRICHOS domain assists in the proper folding and stabilization of SP-C, preventing the formation of toxic aggregates in the alveoli and enabling its normal biological function [76]. Previous research has found that mutations in the *SFTPC* gene are associated with several familial and sporadic interstitial lung diseases, including pulmonary fibrosis [102–104]. These mutations can lead to abnormalities in the structure and function of the SP-C, affecting the composition and function of pulmonary surfactant, ultimately resulting in lung tissue damage and fibrosis [76]. Several studies have linked mutations in *SFTPC* within the BRICHOS domain of the proprotein (proSP-C) to interstitial lung diseases [101, 105]. For example, DNA sequence analyses of the *SFTPC* in children with nonspecific interstitial pneumonia and adults with usual interstitial pneumonia have identified a common heterozygous mutation in exon 5 [106]. This mutation causes a Leu188 to Gln188 change in the carboxy-terminal region of SP-C, potentially impacting peptide processing [106]. These observations suggest that individuals with this specific mutation in the *SFTPC* may have an increased risk of various types of interstitial lung diseases [106]. Using SP-CΔexon4 as a model molecule, a study provides evidence supporting the concept that misfolded BRICHOS mutant isoforms could induce cell injury by disrupting two separate but mechanistically linked subcellular systems, the unfolded protein response (UPR) and the ubiquitin-proteasome system (UPS) [101]. In summary, mutations occurring in the BRICHOS domain of SP-C have been associated with interstitial lung diseases, including pulmonary fibrosis. These mutations can lead to abnormal protein structure and function, affecting the composition and function of pulmonary surfactant, and contributing to lung tissue damage and fibrosis [76, 105, 107–109].

Moreover, our investigation demonstrates that a mutation at position 123 of the *SFTPC* gene, situated within the BRICHOS domain in whales, induces an upregulation of the lung fibrosis marker proteins MMP7 and α-SMA. Comparative analysis reveals heightened MMP7 protein expression in whales compared to wild-type mice. Rescue experiments further indicate increased MMP7 expression in mutant mice compared to their wild-type counterparts, whereas in whale mutants, MMP7 expression is reduced compared to wild-type mice. Similarly, α-SMA protein expression is elevated in whales relative to wild-type mice, with no increase observed in mutant mice compared to wild-type. Consistent with previous research, it is recognized that heightened MMP7 and α-SMA expression contributes to the advancement of pulmonary fibrosis [43, 72, 110]. Building upon prior research, we have identified that marine mammals’ pulmonary surfactant significantly enhances the reduction of alveolar surface tension [33]. This enhancement is vital for alveolar re-expansion during the ascent phase of diving, effectively mitigating the risk of lung squeeze injuries due to alveolar collapse [22, 32, 33]. Additionally, our research elucidates the molecular role of SP-C in pulmonary fibrosis development. This insight not only corroborates the evolutionary adaptation of the lung phenotype in marine mammals but also suggests that the copious, loosely organized elastic fibers in their lungs play a crucial role not only in facilitating alveolar collapse during diving but also in supporting lung expansion upon ascent [22]. In both wild-type and mutant mice, the expression levels of α-SMA protein vary. We believe that amino acid substitutions may have varying effects on protein stability and function in different organisms, and can also affect interactions with other proteins, thereby leading to differences in protein expression levels [111]. The abnormal excessive apoptosis of alveolar epithelial cells is one of the important characteristics of the development of COPD [112, 113]. Therefore, we further investigate the levels of cell proliferation and apoptosis. The cell proliferation assay indicated that at 24 and 48-hour intervals, the proliferation of cetacean wild-type and mutant A549 cells showed no significant change compared to the blank control and murine wild-type and mutant variants. However, apoptosis assays revealed an increase in Cleaved-caspase 3 expression in both wild-type and mutant groups of cetaceans and mice compared to the control, indicating apoptosis occurred in all experimental groups. Notably, more apoptosis was observed in the murine wild-type cells compared to the cetacean mutant variants. Our findings further corroborate previous discoveries that pulmonary fibrosis-induced damage to alveolar epithelial cells leads to an increase in apoptosis levels, while not affecting proliferation levels [114, 115]. Compared to the wild-type cells of mice, there is a reduced level of apoptosis in the wild-type cells of whales. We hypothesize that this could be attributed to adaptive mechanisms evolved by cetaceans due to their prolonged underwater lifestyle. These mechanisms, occurring alongside pulmonary fibrosis in whales, reduce the damage to alveolar epithelial cells, thereby facilitating their respiratory function during diving activities. These results suggest that marine mammals, including whales, have adapted to their long-term aquatic environment by evolving a distinctive phenotype in their lungs. This phenotype is characterized by a marked thickening of the alveolar walls and an abundance of loosely distributed elastic fibers [22, 27].

## Conclusions

To elucidate the molecular mechanisms employed by cetaceans to mitigate the risk of DCS during deep dives, we conducted a comprehensive investigation into 42 lung fibrosis-related genes across representative mammalian lineages. Our findings unveil accelerated evolution, specific amino acid substitutions, and positive selection in multiple lung fibrosis-related genes within cetaceans. This suggests an evolution in their respiratory systems, facilitating diving and minimizing the risk of DCS. Convergent positive selection and amino acid substitutions were also observed in marine mammals. Combined with in vitro experimental validation of the *SFTPC* gene, these results indicate the contribution of lung fibrosis genes to the capacity of marine mammals to reduce the risk of DCS during diving. Moreover, we observed a notable correlation between the rate of gene evolution and diving depth, which reinforces the *selective gas exchange* hypothesis in marine mammals at a molecular scale [13, 14]. This hypothesis elucidates the mechanism by which cetaceans minimize the absorption of N2 during dives. They achieve this through a specialized lung structure featuring collapsed alveolar regions, coupled with the regulation of blood flow to these areas, thereby enabling selective O2 and CO2 exchange with minimal nitrogen exchange [13, 14]. In conclusion, the evolutionary patterns of lung fibrosis-related genes provide novel insights into lung morphology in marine mammals. This research not only enhances our understanding of the molecular evolutionary adaptations of cetaceans to aquatic life but also holds promise for developing strategies to prevent and treat DCS in humans.

### Electronic supplementary material

Below is the link to the electronic supplementary material.

Supplementary Material 1

Supplementary Material 2

## Data Availability

Orthologous sequences of 42 pulmonary fibrosis related genes from 45 species and tree of species used in subsequent evolutionary analysis are available in the figshare Dataset (https://doi.org/10.6084/m9.figshare.24953628.v1). The data generated and analyzed during this study are included in this article and its additional files, including 11 tables and 10 figures.

## Abbreviations

REGs: Rapidly evolving genes
PSGs: Positively selected genes
COPD: Chronic obstructive pulmonary disease
DCS: Decompression sickness
SFTPC: Surfactant Protein C
PAML: Phylogenetic analysis by maximum likelihood
BLAST: Basic local alignment search tool
PGLS: Phylogenetic generalized least squares
LRT: Likelihood ratio test
FDR: False discovery rate
GO: Gene Ontology
