# Supplementary material for: Evolutionary genetics of pulmonary anatomical adaptations in deep-diving cetaceans
Source: BMC Genomics. 2024 Apr 4;25:339. doi: 10.1186/s12864-024-10263-9 (PMC10993460; doi:10.1186/s12864-024-10263-9)
Supplement: Supplementary file 1 — Supplementary Material 1 [file 12864_2024_10263_MOESM1_ESM.docx]

**Evolutionary genetics of pulmonary anatomical adaptations in deep-diving cetaceans**

Boxiong Guo, Yixuan Sun, Yuehua Wang, Ya Zhang, Yu Zheng, Shixia Xu, Guang Yang & Wenhua Ren*

Jiangsu Key Laboratory for Biodiversity and Biotechnology, College of Life Sciences, Nanjing Normal University, Nanjing, China.

^*^Corresponding author: E-mail: 08162@njnu.edu.cn.

**Supplementary Figures**

**
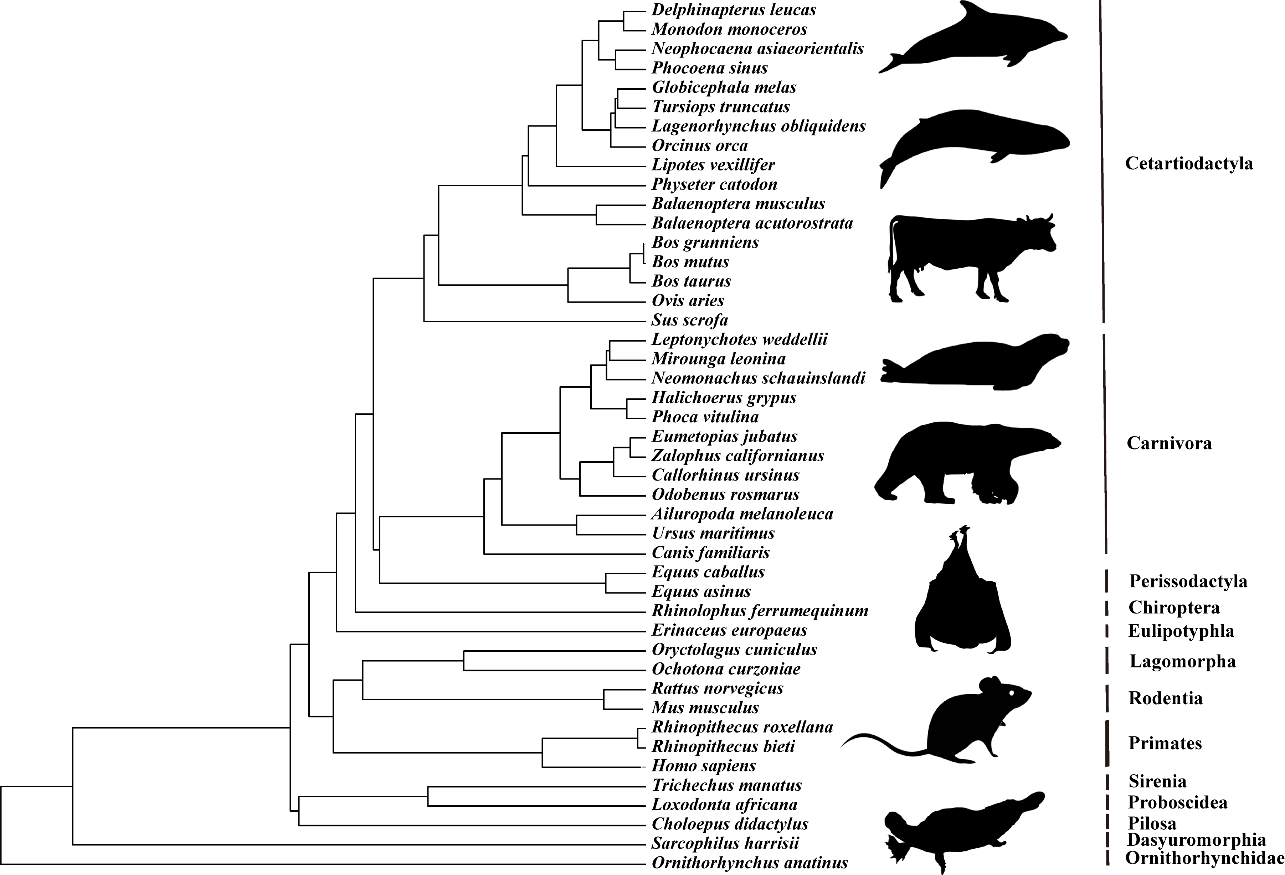
**

**Fig. S1.** The phylogeny of 45 mammals used in PAML (and in other subsequent evolutionary analyses such as PGLS) using TimeTree (<http://www.timetree.org/>), silhouette images come from the PhyloPic (<https://www.phylopic.org/>).

**
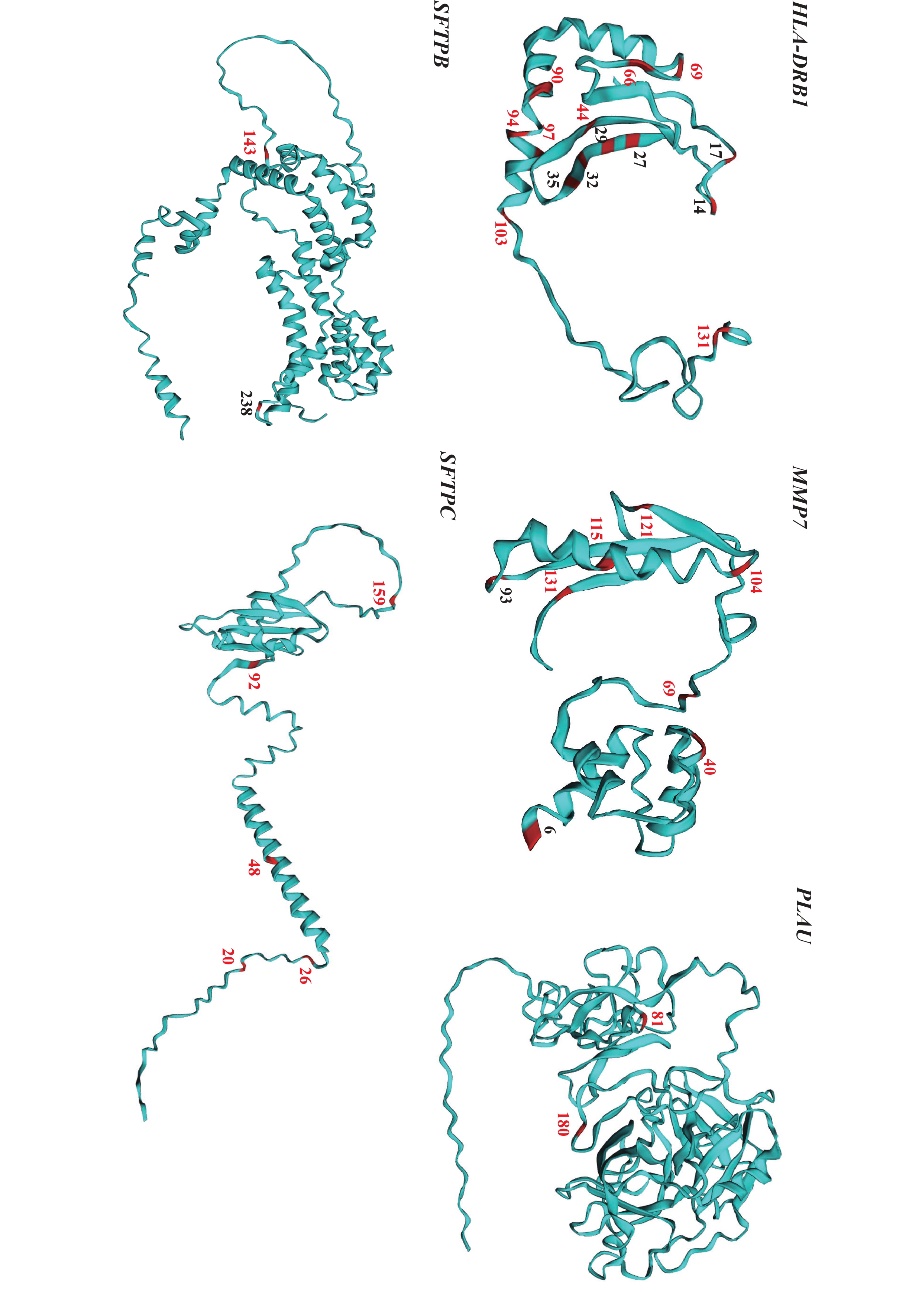
**

**Fig. S2.** The positively selected sites obtained using the branch-site model are mapped onto the three-dimensional structure of the gene. The sites with red numbers are located in the important functional domains of the gene. The figures were created using EzMOL.

**
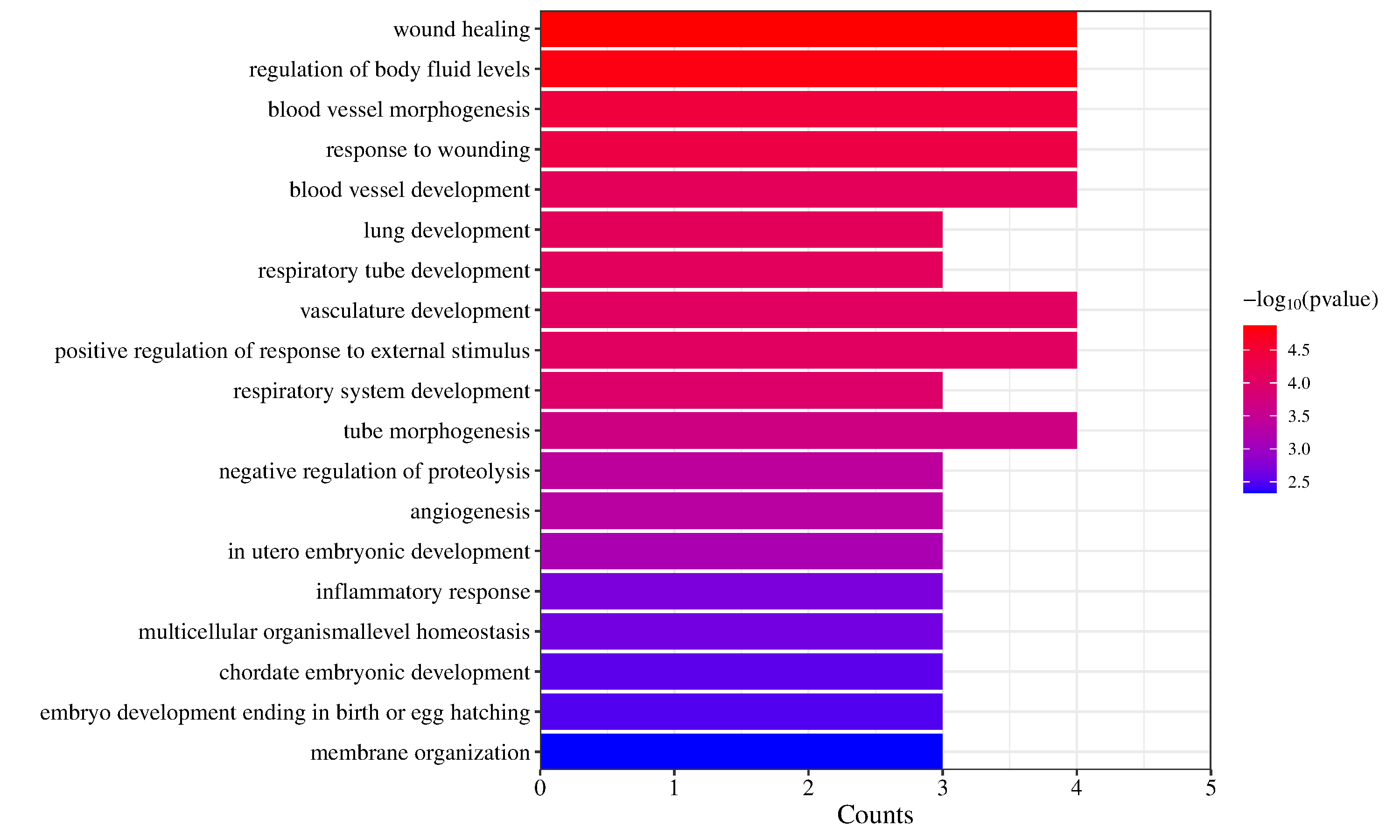
**

**Fig. S3.** GO enrichment terms for fourteen specific amino acid substitutions protein in cetaceans. The 20 related terms in biological process are shown.

**
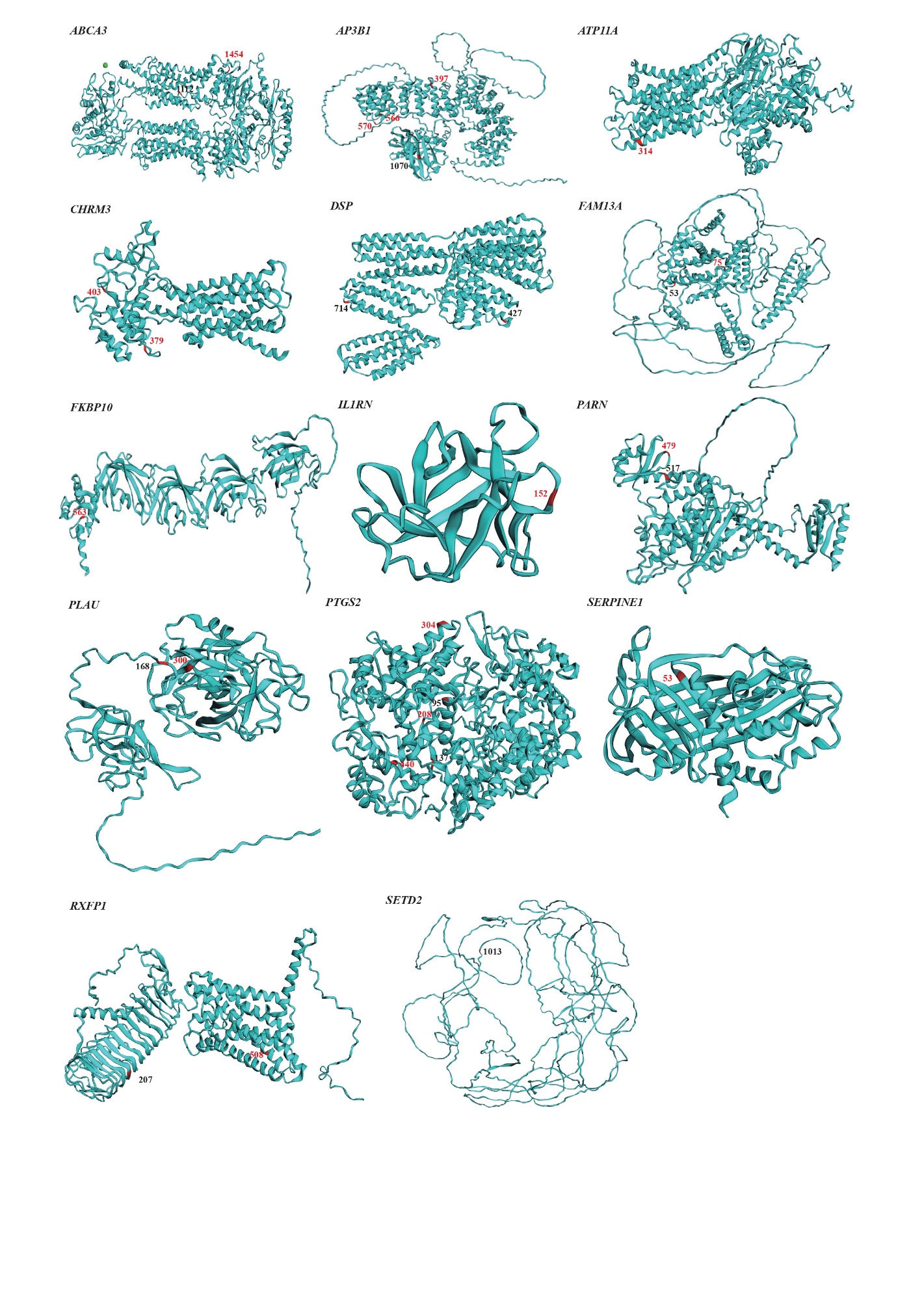
**

**Fig. S4.** cetacean-special amino acid substitutions mapped on the three-dimensional structure of genes. The sites with red numbers are located in the important functional domains of the gene. The figures were created using EzMOL.

**
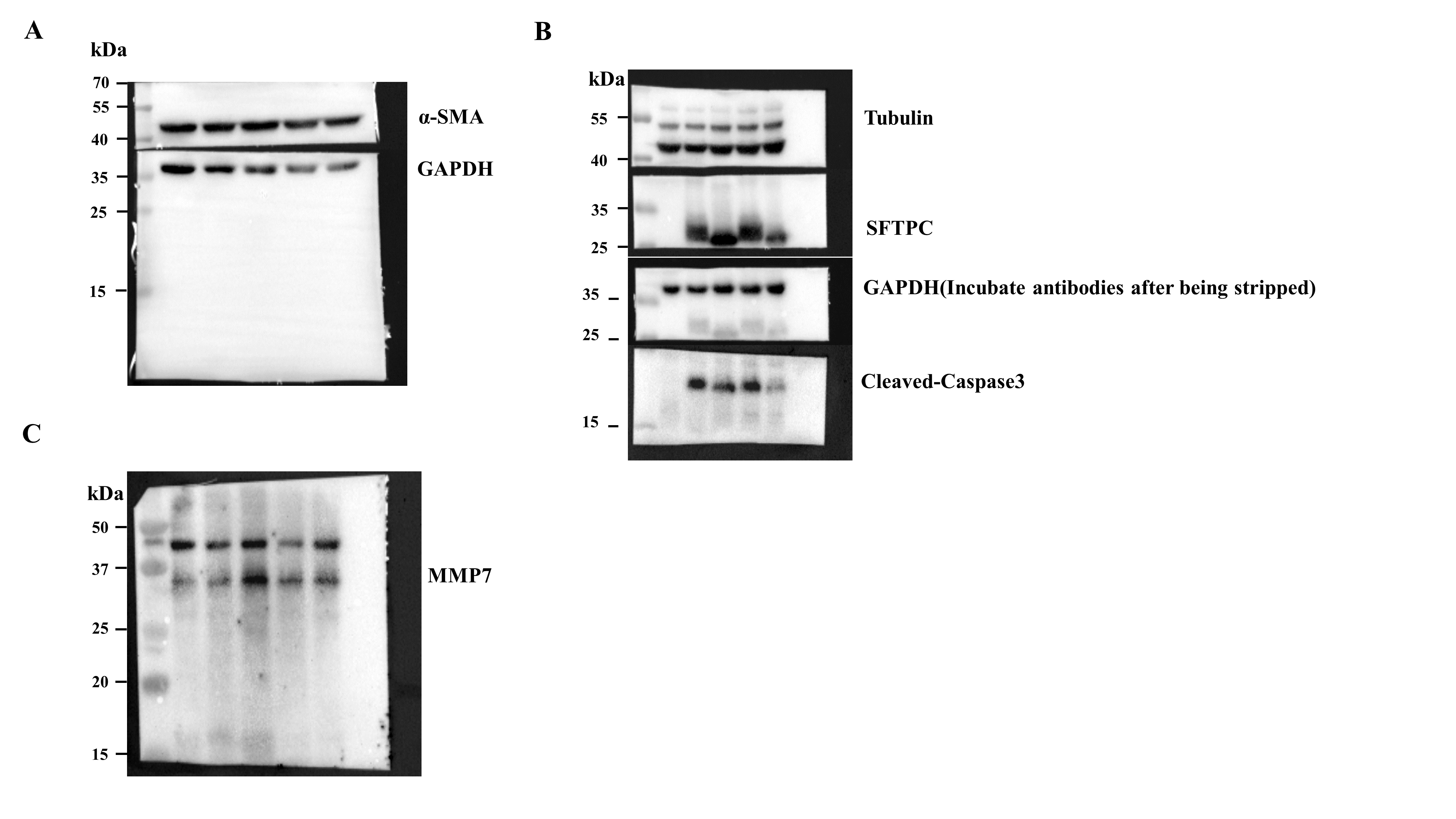
**

**Fig. S5.** Full-length blots/gels of α-SMA, GAPDH (A); Tubulin, SFTPC, GAPDH (Incubate antibodies after being stripped), Cleaved-Caspase 3 (B) and MMP7 (C).
